# The cognitive impairments of electroconvulsive therapy

**DOI:** 10.1192/j.eurpsy.2023.2170

**Published:** 2023-07-19

**Authors:** M. E. G. Mellaci, E. G. Mellaci, M. E. B. Vieira

**Affiliations:** 1 Universidade Nove de Julho, Lins; 2 Universidade Nove de Julho, Bauru, Brazil

## Abstract

**Introduction:**

Electroconvulsive therapy (ECT) is one of the most controversial treatments in medicine, mainly because of its still unknown mechanism of action and uncertainty about cognitive side effects. ECT is used mainly when antidepressant medications do not result in an adequate response in severe depression, and may also be indicated for other disorders. During the acute phase after electroconvulsive therapy there are better cognitive outcomes with unilateral ECT compared to bilateral ECT.

**Objectives:**

This literature review aims to analyze the validity of electroconvulsive therapy despite the cognitive impairment and effectiveness of electroconvulsive therapy.

**Methods:**

The method used was a literary review based on articles published in Scielo and Pubmed. Eighteen electronic articles in English were used in a 10-year time frame, using the term electroconvulsive therapy as descriptors in the title, as well as cognitive impairment in all fields.

**Results:**

We observed that older individuals benefit the most from ECT, with faster response rates and higher remission rates in rapid responders, on the other hand they have a higher risk of cognitive side effects induced by electroconvulsive therapy. Anterograde and retrograde amnesia usually resolve within months. Recovery from retrograde amnesia may be incomplete, resulting in permanent amnesia for events that occurred close to the time of ECT. Right-sided or bilateral electroconvulsive therapy was more effective in treating depression than left-sided. Patients who used right unilateral electroconvulsive therapy had less impairment of verbal memory and post-ictal recovery and faster reorientation compared to left unilateral and bilateral, whereas left unilateral ECT showed a smaller decline in visual memory and non-verbal tests. Bilateral ECT is more effective and faster acting, but is associated with more cognitive impairments compared to unilateral ECT.

**Conclusions:**

We can conclude that ECT is an effective, safe and tolerable treatment, which results in faster and higher remission rates compared to treatment alone with pharmacotherapy. Cognitive effects are largely transient, usually ending within three months. The decision to use unilateral right, left, or bilateral ECT should be made individually for the patient and should be based on a careful assessment of the relative priorities of efficacy versus minimizing cognitive impairment.

**Disclosure of Interest:**

None Declared

